# Europium Nanoparticle-Based High Performing Immunoassay for the Screening of Treponemal Antibodies

**DOI:** 10.1371/journal.pone.0084050

**Published:** 2013-12-26

**Authors:** Sheikh M. Talha, Jukka Hytönen, Adam Westhorpe, Sushil Kumar, Navin Khanna, Kim Pettersson

**Affiliations:** 1 Department of Biotechnology, University of Turku, Turku, Finland; 2 Department of Medical Microbiology and Immunology, University of Turku, Turku, Finland; 3 University of Surrey, Guildford, Surrey, United Kingdom; 4 Recombinant Gene Products Group, International Centre for Genetic Engineering & Biotechnology, Aruna Asaf Ali Marg, New Delhi, India; University of Alabama at Birmingham, United States of America

## Abstract

*Treponema pallidum* subspecies *pallidum* (Tp) is the causative agent of syphilis which mainly spreads through sexual contact, blood transfusion and perinatal route. In order to curtail the spread of the infection and to clinically manage the disease, timely, accurate and reliable diagnosis is very important. We have developed an immunoassay for the detection of treponemal antibodies in human serum or plasma samples. *In vivo* biotinylated and non-biotinylated versions of the recombinant antigen were designed by the fusion of three Tp-specific antigens namely Tp15, Tp17 and Tp47. These fusion antigens were expressed in *E. coli* and purified using single-step metal affinity chromatography. Biotinylated fusion antigen immobilized on streptavidin coated plate was used to capture the treponemal antibodies and the non-biotinylated antigen coated on europium nanoparticles was used as tracer. Assays with two different incubation times of 10 min and 1 h were developed, and following the incubation the europium fluorescence was measured using time-resolved fluorometry. The developed time-resolved fluorometric (TRF) immunoassays were evaluated with in-house and commercial serum/plasma sample panels. For well-established treponemal antibodies positive or negative samples, the sensitivity of TRF immunoassay with 10 min incubation time was 97.4%, and of TRF immunoassay with 1 h incubation time was 98.7%, and the specificities of both the TRF immunoassays were 99.2%. For the samples with discordant results with the reference assays, both the TRF immunoassays showed better specificity than the Enzygnost syphilis enzyme immunoassay as a screening test. The two different incubation times did not have any significant effect on the signal to cutoff (S/Co) ratios obtained with the two immunoassays (p = 0.06). Our results indicate that the developed immunoassay with a short incubation time of 10 min has the potential to be used in clinical laboratories and in blood-bank settings as a screening test for treponemal antibodies.

## Introduction

*Treponema pallidum* subspecies *pallidum* (Tp), a spirochete bacterium, is responsible for syphilis which is a sexually transmitted infection in humans. In 2008 there were an estimated 10.6 million new cases of syphilis in adults worldwide [1]. Worldwide nearly 1.4 million pregnant women, with the disease burden of 44% in Asia and 39% in Africa, had active syphilis infection and were at the risk of transmitting the disease to their unborn babies [2]. Syphilis infection is well known for its multiple stages of pathogenicity, split by stages of latency. As the stages of infection proceed, so does the complication of the disease, thus it is essential that the infection is diagnosed and treated as early as possible [3]–[6]. Syphilis is also one of the transfusion-transmissible infections, which is of a particular concern for the developing countries. To curtail the spread of syphilis through the transfusion of blood and blood-products meticulous screening of the donated blood is paramount [7]–[9].

Serological diagnosis of syphilis includes the detection of two different kinds of molecules, namely treponemal and non-treponemal antibodies. Treponemal antibodies are raised against specific antigens of Tp, and non-treponemal antibodies are raised against cardiolipin [10]. Treponemal antibodies last throughout the life, whereas non-treponemal antibodies are present only in an on-going infection, thus non-treponemal antibody tests are useful to distinguish between convalescent cases and active infections. However, non-treponemal antibody tests usually suffer from low specificity, thus a combination of treponemal and non-treponemal antibodies tests are required for the clinical diagnosis of syphilis. According to WHO's recommendation, in a blood-bank setting screening of only treponemal antibodies should be performed in a population with low incidence of syphilis [11]. Three Tp membrane proteins Tp15, Tp17 and Tp47, named after their respective sizes in kDa, are known to be highly immunogenic, and varying titers of the (treponemal) antibodies against these proteins can be detected in individuals during primary, secondary and latent stages of syphilis infections [12]–[16]. We have developed a double antigen sandwich immunoassay for the detection of treponemal antibodies using a recombinant fusion protein of Tp15, Tp17 and Tp47, and europium nanoparticle. The developed immunoassay is a third generation assay for the detection of total treponemal antibodies (IgM and IgG) intended to be used in clinical laboratories and in blood-bank settings as a screening test. The developed immunoassay was evaluated using in-house and commercial serum/plasma sample panels, and manifested high degrees of sensitivity and specificity.

## Materials and Methods

### Ethics statement

All samples of the in-house human serum panel were collected with informed consent from individuals who might have syphilis in order to perform serologic tests as routine clinical practice. All of these samples were coded samples and strict anonymity was maintained throughout this study. According to the Finnish Medical Research Act (No. 488/1999), Chapter 1, Sections 1, 2 and 3, the research of the present study is not medical research, and thus, it was not necessary to obtain a separate approval from the local Ethics Committee to use the samples in the syphilis serology assays of the present study.

### Generation of recombinant Tp antigens

The synthetic gene, codon-optimized for *E. coli* expression was designed and custom-synthesized from Geneart (Regensburg, Germany), and used to express two versions (biotinylated and non-biotinylated) of the encoded recombinant (r-) Tp antigen. The r-Tp antigen, henceforth termed as Tp15-17-47, was a designer fusion antigen of three Tp membrane proteins namely, Tp15, Tp17 and Tp47, respectively, with flexible tetra-glycyl (G_4_) residues expressing in between the three proteins. This synthetic gene was inserted into pET-32a(+) at *Bam*HI/*Sal*I restriction sites, in-frame with the vector-encoded thioredoxin (Trx) gene and 6x-His tag encoding sequence to express r-Trx-Tp15-17-47 (henceforth termed as r-Tp15-17-47), and into pTrx-BAP vector [17], [18] in-frame with the vector encoded Trx (thioredoxin)-6x-His tag-BAP (biotin acceptor peptide) sequences at the amino terminus, to express the r-Trx-BAP-Tp15-17-47 (henceforth termed as r-Bio-Tp15-17-47) antigen. The resultant plasmids were transformed separately into *E. coli* BL21(DE3) cells, and induced to express with 1 mM IPTG (isopropyl-β-d-thiogalactopyranoside; Qiagen, Hilden, Germany). In order to achieve biotinylation of the r-Bio-Tp15-17-47 antigen *in vivo*, *E. coli* cells expressing this antigen were co-transformed with IPTG-inducible biotin ligase expressing plasmid pBirA [17] and cultured in medium supplemented with biotin (10 µg/ml). After expression of the antigens at 16°C, they were purified from induced cells under denaturing conditions. Briefly, induced cell pellets from 1-L shake flask culture were re-suspended in lysis buffer (20 mM Tris, 8 M Urea, 300 mM NaCl, 10 mM β-mercaptoethanol, 10% glycerol, 20 mM imidazole, pH 8.0) and lysed by sonication at 4°C (Sonics Vibracell sonicator). The resulting lysates were clarified by centrifugation and chromatographed on 5 ml Ni-NTA super flow resin (Qiagen, Hilden, Germany) columns. After washing the columns with the lysis buffer, they were washed with wash buffer (20 mM Tris, 8 M urea, 300 mM NaCl, 10 mM β-mercaptoethanol, 10% glycerol, 20 mM imidazole, 1% Tween-20, pH 8.0) and the elutions were performed using linear gradients of imidazole (20–500 mM) in wash buffer. All the eluted fractions were analyzed by SDS–PAGE, the pure homogeneous fractions were pooled together, and treated with 50 mM DTT and 10 mM EDTA at 37°C for 3 h, and dialyzed extensively against 10 mM Tris, 100 mM NaH_2_PO_4_, 1 mM EDTA, pH 8, at 4°C. The dialyzed proteins were centrifuged, glycerol was added to them up to a final concentration of 10%, and the resulting proteins were concentrated to ∼1 mg/ml, filtered with 0.22 µm membrane and stored at −20°C, until further use.

### Preparation of recombinant Tp antigen coated on europium nanoparticles

The Eu^3+^ chelate-doped Fluoro-Max™ polystyrene nanoparticles (Seradyn Inc., Indianapolis, IN) (107 nm in diameter, >30 000 chelates per particle) have been described before [19], [20]. The buffer of r-Tp15-17-47 was changed to 50 mM sodium carbonate buffer, pH 9.6 using Nucleic Acid Purification (NAP) Sephadex G-25 columns (GE Healthcare, Uppsala, Sweden). The coating of r-Tp15-17-47 on Eu-nanoparticles was performed essentially as described before [18]. r-Tp15-17-47 coated Eu-nanoparticles were stored in 2 mM Tris-HCl, pH 9.0 supplemented with 0.1% BSA and 0.01% sodium azide at 4°C, covered from light. Before every use particles were vortexed thoroughly and sonicated to disperse any large aggregates.

### Serum/plasma samples and their characterization

A collection of 311 human serum/plasma samples was used in this study. This consisted of one in-house panel and two commercially procured panels. The in-house panel of 285 serum samples–where some of the samples were follow-ups from the same patient–was collected at Department of Medical Microbiology and Immunology, University of Turku, Turku, and characterized using the three reference assays i.e. Enzygnost Syphilis competitive enzyme immunoassay (EIA) (Siemens Healthcare Diagnostics Products GmbH, Marburg, Germany) and *Treponema pallidum* haemagglutination assay (TPHA) (Cellognost-Syphilis H, Siemens Healthcare Diagnostics Products GmbH) for the detection of treponemal antibodies, and Oxoid VDRL (Veneral disease research laboratory) carbon antigen agglutination test (Oxoid, Basingstoke, UK) for the detection of reagin (cardiolipin) antibodies. Enzygnost syphilis EIA was used as primary screening test; only the positive samples in this test were tested further with TPHA and VDRL. Samples that appeared non-conclusive with these three reference assays were tested further with Inno-Lia Syphilis score line immunoassay (Innogenetics NV, Gent, Belgium). The two commercial panels, Syphilis Mixed Titer Performance Panel (PSS202) and Syphilis Qualification Panel (QSS701) (SeraCare Life Sciences, Milford, MA, USA) were characterized by the panel supplier using the commercially available assay kits.

### In-house TRF immunoassays for the screening of treponemal antibodies

r-Bio-Tp15-17-47 antigen (200 ng/100 µl/well) was immobilized on streptavidin-coated low-fluorescence microtiter wells (Kaivogen Oy, Turku, Finland) in the assay buffer (50 mM sodium carbonate, pH 9.6, 25 mM NaCl, 2.5% BSA, 1.25% sucrose, 0.06% γ-globulins from bovine blood, 0.1% Tween-20, 0.05% NaN_3_) by incubating it for 1 h at RT with shaking. The wells were then washed twice with wash buffer (50 mM potassium phosphate buffer, pH 7.2, 154 mM NaCl, 0.5 M KCl, 0.1% Tween-20). The antigen-coated wells were used in two TRF immunoassay formats with two different incubation times of 10 min and 1 h. For TRF immunoassay with 10 min incubation time, 25 µl diluted serum/plasma sample (1∶5 in assay buffer) was added to each well, then 25 µl assay buffer containing 2×10^8^ Eu-nanoparticles was added, and incubated for 10 min at RT with shaking. For TRF immunoassay with 1 h incubation time, after adding diluted serum/plasma sample as above, 25 µl assay buffer containing 5×10^7^ Eu-nanoparticles was added, and incubated for 1 h at RT with shaking. After the incubation, the wells were washed 6 times with wash buffer. Time-resolved fluorescence for europium was measured (λ_ex_: 340 nm; λ_em_: 615 nm) from dry wells using Victor^3^V 1420 Multilabel counter (modified standard europium protocol; measurement height 5 mm).

### Statistical analysis

Samples were designated as either positive or negative using cutoff values of 1203 counts per second (cps) and 1212 cps, respectively, for the TRF immunoassays with 10 min and 1 h incubation times. These cutoff values were obtained by adding 4 times the standard deviation (SD) to the mean read-out of 119 Tp-negative samples (all the samples of category #6, Table 1, except one sample which was repeatedly positive with the TRF immunoassays) of the in-house panel in the TRF immunoassays with 10 min incubation (mean = 321 cps; SD = 221 cps) and in 1 h incubation (mean = 308 cps; SD = 226 cps) time as described above. Serum samples with ‘signal-to-cutoff’ (S/Co) ratios of <1.0 were designated as negative, while those with S/Co≥1.0 were designated as positive.

**Table 1:** Evaluation summary of the TRF immunoassays using the in-house serum sample panel.

		Reference assays^a^			TRF immunoassays^a^	
Category #	No. of samples (n = 285)	Enzygnost Syphilis	TPHA	VDRL/Oxoid	10 min incubation	1 h incubation
1	56	56/56	56/56	56/56	56/56	56/56
2	73	73/73	73/73	0/73	69/73	71/73
3	27	27/27	0/27	0/27	10/27	10/27
4	8	8 (+/−)/8	0/8	0/8	0/8	0/8
5	1	1/1	0/1	1/1	1/1	1/1
6	120	0/120	ND	ND	1/120	1/120

^a^ Results expressed as ‘number of positive samples with the indicated assays’/‘total number of samples’. ‘+/−’ indicates that indeterminate result was obtained with the respective assay; ‘ND’ indicates that the test was not done.

Statistical significance of differences in sensitivities between the two TRF immunoassays was determined using two-tailed paired t-test. Differences were considered to be of statistical significance when the probability (p) was <0.05.

## Results

### Design, expression and purification of the recombinant antigens

**Figure 1:**
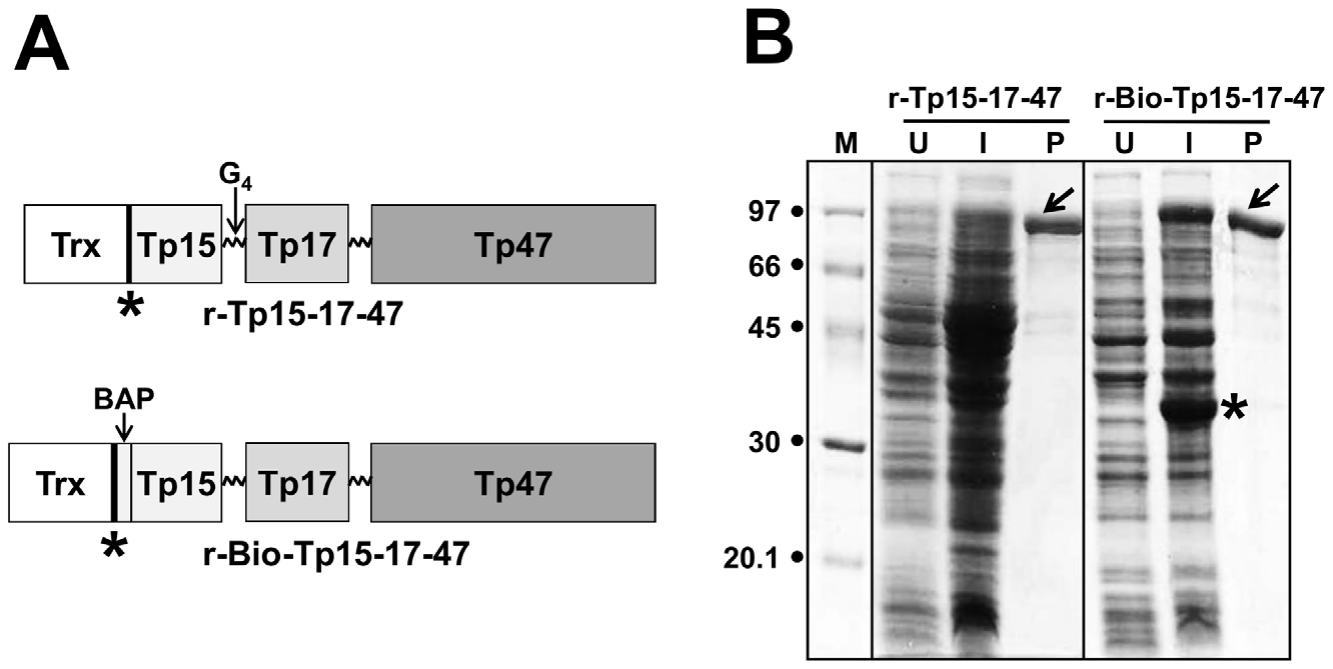
Design, expression and purification of the r-antigens used in the study. (A) The r-Tp15-17-47 antigen is a chimeric in-frame fusion construct containing thioredoxin, 6x His tag (indicated by the asterisk), followed by the membrane proteins (Tp15, Tp17 and Tp47) of Tp stitched together with flexible tetra-glycyl (G_4_) linkers (presented with zig-zag lines). In r-Bio-Tp15-17-47, the BAP is inserted in-frame to generate the *in vivo* biotinylated version of the antigen. (B) SDS-PAGE analysis of r-Tp15-17-47 and r-Bio-Tp15-17-47 antigens. Aliquots of total cell lysates of *E. coli* harboring the r-p15-17-47 and r-Bio-p15-17-47 antigen constructs (*uninduced*: lanes marked ‘U’; *induced*: lanes marked ‘I’), and aliquots of the affinity-purified dialyzed and soluble antigens (lanes marked ‘P’), were electrophoresed on denaturing gels and visualized by Coomassie staining. Protein size markers were run in lane ‘M’; their sizes (in kDa) are shown on the left. The arrows shown denote the bands of purified proteins. The asterisk denotes the position of biotin ligase enzyme.

The design of the r-Tp15-17-47 antigen and its *in vivo* biotinylated version, r-Bio-Tp15-17-47 is shown in Figure 1A. r-Tp15-17-47 antigen was genetically engineered to express as an in-frame fusion with Trx and a 6x-His tag at its amino terminus to facilitate its solubility and purification. The *in vivo* biotinylated version also included BAP sequence. In order to efficiently *in vivo* biotinylate the antigen, r-Bio-p15-17-47 was co-expressed with biotin ligase enzyme in the media supplemented with biotin. Both the antigens were produced starting from a 1-L shake-flask culture of *E. coli*. The lysed cells were analysed before and after induction by SDS-PAGE. Typical expression profiles of the clones are shown in Figure 1B. The induced cell lysates harbouring the r-Tp15-17-47-expressing plasmids did not clearly show the distinct over-expression of a new polypeptide of the expected molecular weight of ∼95 kDa, which is the calculated size of r-Tp15-17-47. The induced cells harbouring the r-Bio-Tp15-17-47-expressing plasmids showed the distinct bands of new polypeptides of ∼97 and ∼34 kDa, which were consistent with the calculated sizes of the r-Bio-Tp15-17-47 and the biotin ligase enzyme, respectively. In both the cases, the un-induced cells did not show the presence of these proteins. Western blot analysis performed with the lysates using a monoclonal antibody specific to the 6x-His tag confirmed the presence of both r-Tp15-17-47 and r-Bio-Tp15-17-47 proteins in their respective induced lysates. However, initial studies revealed that in the native conditions both the proteins did not bind to the Ni-NTA resin (data not shown). We speculated that this was due to the inaccessibility of 6x-His tag to bind to the resin. To circumvent this problem urea was used to make the 6x-His tag accessible and the Ni-NTA affinity purifications of the antigens were carried out under denaturing conditions. Purified proteins were treated with DTT and EDTA, and dialyzed to remove the denaturants, and soluble proteins were analysed by SDS-PAGE (Figure 1B). At least 25 mg of each of the soluble purified proteins were obtained from 1-L induced *E. coli* culture.

### In-house TRF immunoassays for treponemal antibodies

**Figure 2:**
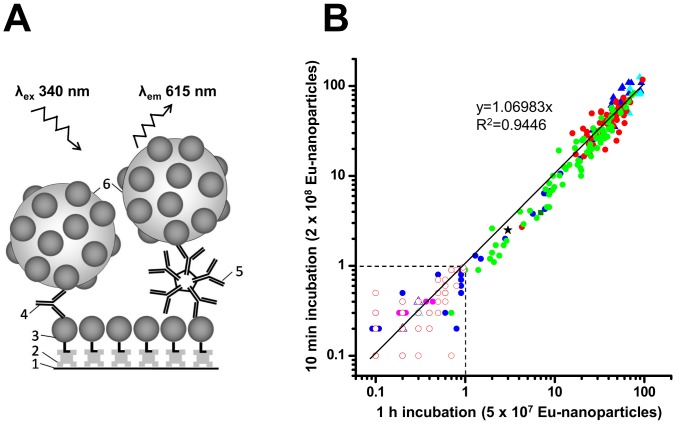
Design and evaluation of the in-house TRF immunoassays. (A) Schematic representation of the in-house TRF immunoassay. The design of both the TRF immunoassays was same, except for different incubation times and tracer amounts. The numbers represent the following: (1) microtiter well surface, (2) streptavidin, (3) r-Bio-p15-17-47, (4) serum anti-Tp IgG antibody, (5) serum anti-Tp IgM antibody, and (6) r-p15-17-47 coated on Eu^3+^ chelate-doped nanoparticles. (B) Scatter plot with the S/Co values of all serum samples (n = 311) analyzed in this study, using the in-house TRF immunoassays with 1 h (*x* axis) and 10 min (*y* axis) incubation times. Different symbols represent positive (+) or negative (-) serum samples, either from commercial panels based on the results provided by the panel supplier, or from six different categories (#1 – #6) of the in-house samples based on their reactivities with the reference assays as described in Table 1. Symbols for different kind of samples are as the following: PSS202 (+), filled blue triangles; PSS202 (-), empty blue triangles; QSS701 (+), filled cyan triangles; QSS701 (-), empty cyan triangle; samples of category #1, filled red circles; samples of category #2, filled green circles; samples of category #3, filled blue circles; samples of category #4, filled magenta circles; sample of category #5, filled olive square; samples of category #6 (except one sample), empty red circles; and exceptional sample of category #6 which gave positive results with in-house TRF immunoassays, black star. Dashed vertical and horizontal lines represent the cutoffs (at S/Co = 1) for the two TRF immunoassays.

After the purification of the pair of recombinant antigens, these antigens were utilized for the development of double antigen sandwich immunoassays for the detection of treponemal antibodies (immunoglobulins). In these immunoassays, the treponemal immunoglobulins (Ig) of both the classes (IgM and IgG classes) from serum/plasma samples were captured on r-Bio-Tp15-17-47 antigens immobilized on streptavidin-coated microtiter wells. The detection of these antibodies was made by r-Tp15-17-47 coated on Eu-nanoparticles, and the fluorescence was measured using time-resolved fluorometry. Two different incubation times and different amount of tracers were used for the two TRF immunoassays; however the design of the both the TRF immunoassays is essentially the same, which is presented in Figure 2A.

The performances of both the TRF immunoassays were evaluated with serum/plasma samples (n = 311) from an in-house panel and two commercially available panels. The samples (n = 285) of in-house panel were divided into six different categories based on their reactivities with the three reference assays (Table 1). Category #1 consisted of 56 samples that belonged to 31 people, and the samples were (sero)positive with all three reference assays. All of these samples were identified as positive in both the in-house TRF immunoassays, without any ambiguity.

Category #2 (Table 1) consisted of 73 samples from 52 people. All of these samples were positive with Enzygnost and TPHA, and negative with VDRL. Out of these 73 samples, two were detected as negative in both the TRF immunoassays, and two as negative in only the TRF immunoassay with 10 min incubation time. The latter two samples had S/Co ratios of 1.4 and 1.0 respectively in TRF immunoassay with 1 h incubation time, and S/Co ratio of 0.9 for both the samples in TRF immunoassay with 10 min incubation time. Only the minor differences in the S/Co ratios that are at or very close to the borderline (S/Co = 1) in these two samples in both the TRF immunoassays, provided the negative results in the TRF immunoassay with 10 min incubation time. Further data analysis revealed that all of these 4 samples had low titers of antibodies and were weak positive samples, as indicated by the Enzygnost and TPHA test results. Three of these 4 samples are most likely to have antibodies due to old immunity. One of these 4 samples, which was negative with both the TRF immunoassays was from a new-born baby. A follow-up of this sample was available and tested in this study (one of the samples in category #4, Table 1; sample serial #13, Table 2), and again detected as negative with both the TRF immunoassays.

**Table 2:** Results from the non-conclusive primary and their respective follow-up samples mostly from category #3 and #4 in Table 1.

	Results from primary samples^a^						Results from follow-up samples^b^							
		Reference assays			TRF immunoassays			Reference assays				TRF immunoassays		
Serial #	No. of samples (n = 25)	Enzg.	TPHA	VDRL	10 min incubation	1 h incubation	No. of sample (n = 13)	Enzg.	TPHA	VDRL	I-L	10 min incubation	1 h incubation	Syphilis status^c^
1	1	+	-	-	-	-	1	+/-	-	-	-	-	-	N
2	1	+	-	-	-	-	NA	NA	NA	NA	NA	NA	NA	P
3	3	+	-	-	+	+	NA	NA	NA	NA	NA	NA	NA	P
4	6	+	-	-	-	-	6	+	-	-	-	-	-	N
5	2	+	-	-	+	+	NA	NA	NA	NA	NA	NA	NA	?
6	1	+	-	-	+	+	1†	+	+(80)*	-	+/-	+	+	P
7	1	+	-	-	-	-	1†	+	+(80)*	-	ND	+	+	P
8	2	+	-	-	+	+	2	+	-	-	+	+	+	P
9	1	+	-	-	-	-	1‡	-	ND	ND	ND	ND	ND	N
10	1	+	-	-	-	-	NA	NA	NA	NA	NA	NA	NA	?
11	1	+/-	-	-	-	-	1	+/-	-	-	-	-	-	N
12	4	+/-	-	-	-	-	NA	NA	NA	NA	NA	NA	NA	?
13	1	+/-	-	-	-	-	NA	NA	NA	NA	NA	NA	NA	N§

^a^ Results obtained from the primary samples using the three reference assays, and both the TRF immunoassays (with 10 min and 1 h incubation times), as indicated. All the primary samples are from category #3 and #4, as listed in Table 1. ‘Enzg.’ indicates Enzygnost syphilis EIA. ‘+’, ‘−’ and ‘+/−’ indicate positive, negative and indeterminate results, respectively, as obtained with the mentioned assays.

^b^ Results obtained from the thirteen follow-up samples of their respective primary samples using Enzygnost syphilis, TPHA, VDRL and I-L (Inno-Lia Syphilis score line immunoassay) as reference assays, and both the TRF immunoassays (with 10 min and 1 h incubation times), as indicated. Ten out of 13 samples are from category #3 and #4, as listed in Table 1. Each primary sample had either one or none follow-up sample. ‘NA’ indicates that a follow-up sample was not available to us.

sample belongs to category #2 as divided in Table 1.

Sample not tested with TRF immunoassays in this study.

*The antibody titer obtained in TPHA assay is shown in parentheses. ‘ND’, indicates that the test was not done.

^c^ Syphilis status indicates whether the person has, or has had syphilis previously, and is based upon the results of previous or follow-up samples and on the available clinical data. ‘N’ indicates a negative and ‘P’ indicates a positive status for syphilis. ‘?’ indicates that true syphilis status is unknown.

sample from a new-born baby, with borderline level of maternal antibodies (data not shown).

Category #3 (Table 1) consisted of 27 samples that were detected as positive with Enzygnost, but negative with TPHA and VDRL. These results with Enzygnost may be false positive, or may be due to old immunity or very early infection. Ten samples of this category were detected as positive and 17 as negative with both the TRF immunoassays. Category #4 (Table 1) consisted of 8 samples that produced indeterminate results with Enzygnost, and negative results with TPHA and VDRL tests. None of these 8 samples were detected as positive in the TRF immunoassays. Category #3 and #4 together consisted of 35 samples from 25 persons. The results obtained using the reference assays and the TRF immunoassays, with these samples and three additional samples have been described in Table 2. None of the primary samples or their follow-ups with a negative syphilis status detected as positive in both the TRF immunoassays, although these samples were detected as positive in Enzygnost syphilis reference test. One sample (serial #2) detected as negative with both the TRF immunoassays, but its syphilis status was positive based on Inno-Lia syphilis test result obtained with the previous sample from the same patient (data not shown). However, a follow-up sample from this patient was not available. One sample (serial #7) was detected as negative, but its respective follow-up sample was detected as positive in both the TRF immunoassays. Other samples and/or their follow-ups with positive syphilis status were detected correctly in both the TRF immunoassays. Two samples with unknown syphilis status were detected as positive, and five samples with unknown syphilis status were detected as negative in both the TRF immunoassays.

Category #5 (Table 1) consisted of a lone sample which was low positive with Enzygnost and VDRL, and negative with TPHA test. A follow-up sample from this person was not available to us. Both the TRF immunoassays identified this sample as positive.

Category #6 (Table 1) consisted of 120 samples that were all negative with Enzygnost assay. These samples were not tested further with TPHA or VDRL reference assays. All of these samples were identified correctly with both the TRF immunoassays, except for one. This lone exceptional sample was detected as positive with both the TRF immunoassays repeatedly. This sample was re-tested with Enzygnost, TPHA and VDRL, and detected as negative with all three reference assays.

The performances of these TRF immunoassays were further evaluated with two commercially available panels. Syphilis Mixed Titer Performance Panel (PSS202) is a panel of naturally occurring plasma samples. The panel consisted of 18 Tp positive and 2 Tp negative samples based on the assays performed for the detection of treponemal and non-treponemal antibodies using the commercial kits, as indicated in Table 3. Both the TRF immunoassays identified all the samples unequivocally. Syphilis qualification panel (QSS701) consists of six samples with established reactivity in syphilis assays. These samples were manufactured from human serum or plasma, and five of them are reactive and one is non-reactive in syphilis assays. Both the TRF immunoassays identified all the samples correctly without any ambiguity (Table 4).

**Table 3:** Evaluation of the TRF immunoassays using Syphilis Mixed Titer Performance Panel (PSS202).

	RPR, result expressed as Titer^a^		ATA, result expressed as S/Co^a^		ATA, result expressed as result^a^		TRF immunoassays, result expressed as S/Co^b^	
Member ID#	Becton-Dickinson	Wampole	Diesse EIA	Trinity EIA	Fujirebio TPPA	Olympus TPHA	10 min incubation	1 h incubation
1	128	128	>5.5	4.4	R	R	98.1 (+)	57.1 (+)
2	8	8	>5.5	3.8	R	R	59.9 (+)	57.3 (+)
3	4	4	>5.5	3.3	R	R	87.7 (+)	91.0 (+)
4	Neg	Neg	0.3	0.3	NR	NR	0.2 (−)	0.2 (−)
5	1	Neg	>5.5	2.2	R	R	66.7 (+)	54.7 (+)
6	64	128	>5.5	4.5	R	R	108.4 (+)	71.5 (+)
7	8	8	>5.5	3.6	R	R	83.0 (+)	67.5 (+)
8	1	1	>5.5	2.3	R	R	32.0 (+)	26.6 (+)
9	32	32	4.5	3.8	R	R	67.0 (+)	44.4 (+)
10	16	16	>5.5	4.4	R	R	93.8 (+)	55.3 (+)
11	4	4	5.1	2.5	R	R	25.2 (+)	26.1 (+)
12	32	64	>5.5	3.9	R	R	109.3 (+)	67.0 (+)
13	1	2	>5.5	3.2	R	R	108.2 (+)	93.0 (+)
14	2	2	>5.5	3.3	R	R	64.4 (+)	53.1 (+)
15	32	64	>5.5	3.5	R	R	75.2 (+)	45.1 (+)
16	Neg	Neg	0.3	0.3	NR	NR	0.4 (−)	0.3 (−)
17	2	2	>5.5	2.4	R	R	61.9 (+)	59.0 (+)
18	1	Neg	>5.5	2.4	R	R	60.7 (+)	42.9 (+)
19	2	2	>5.5	2.2	R	R	35.2 (+)	46.7 (+)
20	Neg	Neg	>5.5	2.2	R	R	53.4 (+)	45.0 (+)

^a^ Assays performed using commercial kits as indicated. Results were provided by the panel supplier. RPR, rapid plasma reagin; ATA, anti-*Treponema* antibody; S/Co, signal to cutoff ratio; EIA, enzyme immunoassay; TPPA, *Treponema pallidum* particle agglutination assay; TPHA, *Treponema pallidum* haemagglutination assay; Neg, negative; R, reactive; NR, non-reactive. RPR results are endpoint dilutions. S/Co ratios ≥1.0 are considered reactive.

^b^ Values indicate S/Co ratios obtained in this study, using the in-house TRF immunoassays with 10 min and 1 h incubation times. Samples with S/Co values <1.0 are designated as negative (−) and those with values ≥1.0 are designated as positive (+).

**Table 4:** Evaluation of the TRF immunoassays using Syphilis Qualification Panel (QSS701).

		TRF immunoassays^c^	
Member ID#^a^	Syphilis reactivity^b^	10 min incubation	1 h incubation
1	R	125.6 (+)	88.8 (+)
2	R	81.0 (+)	88.3 (+)
3	R	83.3 (+)	91.3 (+)
4	R	49.6 (+)	68.6 (+)
5	R	93.7 (+)	69.3 (+)
6	NR	0.3 (−)	0.3 (−)

^a^ Members of the panel are manufactured from human serum or plasma, as provided by the panel supplier. Five members are formulated with various reactivities of Syphilis. Non-reactive member was formulated from Syphilis non-reactive pools.

^b^ Results were provided by the panel supplier. R, reactive; NR, non-reactive.

^c^ Values indicate S/Co ratios obtained in this study, using the in-house TRF immunoassays with 10 min and 1 h incubation times. Samples with S/Co values <1.0 are designated as negative (−) and those with values ≥1.0 are designated as positive (+).

The sensitivity and specificity of both the TRF immunoassays were calculated by using the samples of the in-house panel and commercial panel that were established as either positive (samples of category #1 and #2 of Table 1, and 18 positive samples of PSS202) or negative (samples of category #6 of Table 1, and 2 negative samples of PSS202) for treponemal antibodies based on the results of the commercial reference assays. The sensitivity of TRF immunoassay with 10 min incubation time was 97.4%, and of TRF immunoassay with 1 h incubation time was 98.7%. The specificities of both the TRF immunoassays were 99.2%.

Data obtained in this study indicated that there is a very good correlation in between the two TRF immunoassays. To delineate this observation more precisely, a scatter plot was drawn to compare the S/Co values obtained for all the samples used in this study in both the TRF immunoassays. The coefficient of determination (R^2^) of the linear regression for the two TRF immunoassays was 0.9446 (Figure 2B). The two TRF immunoassays produced proximate S/Co values for most of the samples (p = 0.06, using paired t-test). Our results indicate that the likelihood of getting similar qualitative results with similar S/Co ratios for the screening of treponemal antibodies in the serum/plasma samples in the two TRF immunoassays is very high.

## Discussion

The diagnosis of syphilis is primarily based on serological immunoassays namely treponemal and non-treponemal tests because, (i) the culture of the bacteria *in vitro* has not been possible so far, (ii) the nucleic acid tests are expensive and not widely available to all of the laboratories, (iii) and direct visualisation of the bacteria requires sophisticated microscopy techniques, which has its own limitations, and is not available in all of the laboratories [21]. Syphilis diagnosis is complex, and there are different algorithms being followed for its diagnosis, namely *traditional algorithm* where the non-treponemal antibodies are first tested with rapid plasma reagin (RPR) or VDRL and the positive results are further confirmed with treponemal antibodies test e.g. TPHA, and *reverse algorithm* in which treponemal antibodies are first tested with an immunoassay, and positive samples are further tested with another treponemal assay e.g. TPHA and with a non-treponemal assay [22], [23]. There are several publications where the arguments have been given for shifting to the reverse algorithm from the traditional one [16], [22]–[24]. Countries like Finland are already following the reverse algorithm, and all the in-house samples used in this study are characterized following this algorithm only. The dependence on serologic assays for the diagnosis of syphilis calls for highly sensitive and specific treponemal and non-treponemal antibodies tests.

In this study we have sought to develop a sensitive immunoassay for the treponemal antibodies that could be used as screening test in clinical laboratories as well as in blood-bank settings. While developing this immunoassay two main aspects were kept in mind namely, affordability and performance. Since Tp15, Tp17 and Tp47 are very immunogenic proteins and show good performance in the diagnosis of treponemal antibodies, we fused these three antigens in tandem using flexible tetra-glycyl linkers to design the fusion protein Tp15-17-47. We used r-Bio-Tp15-17-47 antigen as capture and r-Tp15-17-47 antigen coated on Eu-nanoparticle as tracer to devise double antigen sandwich or bridge TRF immunoassays with two different incubation times of 10 min and 1 h. Both the TRF immunoassays were evaluated with in-house and commercial serum/plasma sample panels. Samples from in-house panel were divided into different categories (#1–#6) (Table 1). All the samples from category #1 were positive with the three reference assays indicating that these samples belonged to the patients who had active or recently treated Tp infections. Patients with active infection need urgent medical treatment in order to avoid any complications and further transmission of infection to others. Both the TRF immunoassays identified these samples correctly indicating their high sensitivities to recognise treponemal antibodies.

All the samples of category #2 were positive only with Enzygnost and TPHA reference assays, indicating that these samples have only the treponemal antibodies that are most likely due to the old immunity or due to very early infection where the levels of reagin antibodies are too low to be detected. The samples of this category that were detected as negative with the TRF immunoassays included a sample from a new-born baby, and a follow-up of this sample was also detected as negative with both the TRF immunoassays. This was likely a case of declining maternal antibodies in the new-born, because it is established that the maternal treponemal IgG may cross the placenta [25]. The individuals belonging to this category are not considered to have an active Tp infection, and are not needed to be given medical treatment. So the samples of this category that were detected as negative by the TRF immunoassays are not clinically relevant.

Samples of Category #3 and #4 are further delineated in Table 2. Two samples (serial #2 and #7) with positive syphilis status were detected as negative with the TRF immunoassays. One sample (serial #2) was given positive syphilis status based on the result obtained from its previous sample; this is most likely a case of resolved Tp infection. Follow-up of sample serial #7 turned positive with both the TRF immunoassays. With this follow-up sample TPHA test provided low positive result, indicating that most likely this was a case of an on-going syphilis infection with seroconverting antibodies. Data from Table 2 indicate that the TRF immunoassays are not likely to produce any false positive results, and are more specific than the Enzygnost syphilis EIA as screening test for treponemal antibodies.

The results obtained for the lone sample of category #5 (Table 1) with the reference assays indicate that perhaps this was a case of very recent primary Tp infection. One sample of category #6 which was detected as positive (S/Co = 2.5 and 3 with TRF immunoassays with 10 min and 1 h incubation times, respectively) with both the TRF immunoassays, might be a case of previous immunity or an acute Tp infection which was not detected by the three reference assays, however a follow-up of this sample was not available to us. The TRF immunoassays were further evaluated with the two commercial panels, and all the samples were unequivocally identified. The overall results obtained in the TRF immunoassays with the in-house and commercial panels indicate that these developed assays are highly sensitive and specific for the detection of treponemal antibodies.

Much higher S/Co values were obtained in both the TRF immunoassays with in-house panel (data not shown) and commercial panel (Table 3) as compared to the respective reference EIAs used. High S/Co values obtained in the TRF immunoassays are attributable to the design of the fusion antigen and the strategy to amplify the signals using Eu-nanoparticles as labels. These antigens were produced in-house using *E. coli* expression system. The advantage of using fusion proteins was a much lower production cost as compared to the production of three different biotinylated and non-biotinylated antigens. A fusion protein also contributes to the higher epitope density of the capture surface, hence increasing the sensitivity of the immunoassay. Using a double antigen sandwich assay approach we have devised third generation immunoassays that can by design detect total treponemal antibodies (IgM and IgG) [26].

Both the TRF immunoassays performed in approximately the same way in identifying the samples correctly, irrespective of the incubation times involved in the two assays. This implies that 10 min incubation time is sufficient for the TRF immunoassay to detect treponemal antibodies. High performance of the TRF immunoassay coupled with a short incubation time makes it suitable for use as a screening test not only in the clinical laboratories where the results need to be produced as soon as possible in order to determine the syphilis status of the patients, but also in blood-bank settings. In-house TRF immunoassay is capable to be automated on high-throughput instrumentations, further making it suitable for use as a screening test in large centralized laboratories. In future, the use of up-conversion-based nanoparticles as reporter that can be detected using a low-cost reader instrument will pave the way for an inexpensive test that can be used without compromising the performance in resource poor laboratories also.
